# Fast-track hip and knee replacement — what are the issues?

**DOI:** 10.3109/17453674.2010.487237

**Published:** 2010-05-21

**Authors:** Henrik Kehlet, Kjeld Søballe

**Affiliations:** Section for Surgical Pathophysiology, Rigshospitalet, Copenhagen University, CopenhagenDenmark; Department of Orthopaedics, Aarhus University Hospital, Aarhus; the Lundbeck Centre for Fast-track Hip and Knee Surgery, CopenhagenDenmark

## Introduction

Surgical injury is followed by pain, stress-induced catabolism, impairment of organ function, and a risk of thromboembolism and impaired cognitive function. These events may contribute to complications, a need for prolonged hospitalization, postoperative fatigue, delayed convalescence, and the need for rehabilitation. Optimization of the individual care components in perioperative care (the fast-track methodology) reduces the need for hospitalization, morbidity, and prolonged convalescence, with subsequent economic savings (Kehlet and Wilmore 2008). Correspondingly, improvement in perioperative care has led to a reduced stay in hospital after total hip arthroplasty (THA) and total knee arthroplasty (TKA), which is now only about 3 days in many centers.

The main question that remains to be answered is whether further improvement can be made and whether these operations can be performed on an ambulatory or semi-ambulatory basis without any increased risk of morbidity or cardiopulmonary and thromboembolic complications, long-term cognitive dysfunction, pain, and need for rehabilitation. Thus, a multidisciplinary collaboration has been established in 5 dedicated public Danish hospitals with established fast-track THA and TKA in order to optimize perioperative care to require only 1–2 days of hospitalization with no increase in post-discharge pain, morbidity, and rehabilitation requirements (Figure). To this end, we concentrated on perioperative pain management, optimization of transfusion strategies, strategies for postoperative rehabilitation, requirements for postoperative thromboembolic prophylaxis, and safety aspects (re-admissions, hip dislocation, and knee stiffness).

Effective postoperative pain relief is a prerequisite for a successful fast-track recovery. Although continuous peripheral nerve blocks are currently the most effective analgesic techniques (Ilfeld et al. 2008a,b), they are demanding in terms of expertise and there is a risk of muscle paralysis. Efforts have therefore been made recently to improve local wound infiltration and infusion techniques. Although promising, more research is required to better define these techniques regarding dose-volume relationships, placement and types of catheters, duration, and safety aspects (Röstlund and Kehlet 2007). There will be more focus on post-discharge pain, which remains a problem in many patients. Furthermore, research will concentrate on mechanisms and predictors of the large inter-individual variability in the degree of postoperative pain, which may be related to the inflammatory response and genes associated with pain. Such knowledge should facilitate pain management, leading to differential use of analgesic principles in low pain responders and high-pain responders.

Blood transfusion is indicated when a patient's limit concerning—or physiological adaptation to—progressive normovolemic anemia is reached, but there are no specific data from THA and TKA. Data from hip fracture surgery have shown that postoperative anemia impedes functional mobility and that a liberal transfusion strategy reduces morbidity (Foss et al. 2009). Since modern blood transfusion is safer than before, large randomized studies with high and low transfusion thresholds will be performed in cardiovascular high-risk patients after THA, with morbidity and functional recovery as primary outcome measures.

**Figure:**
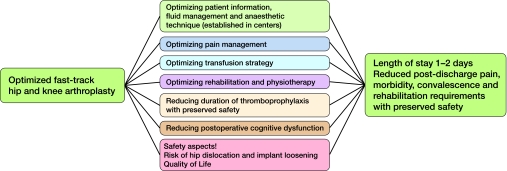


Postoperative rehabilitation after THA and TKA has been standard despite the fact that there has been little evidence on the optimal type of physiotherapy, when it should be started, and for how long (Minns Lowe et al. 2007, 2009). Previous randomized studies have been inconclusive and they were based on relatively long-term hospitalization (7–12 days); they are therefore not applicable to fast-track THA and TKA.

Over the last decade, many industry-sponsored trials have documented the need for prolonged thromboembolic prophylaxis (10–30 days), mostly based on surrogate outcomes and with relatively small effects on clinical manifestations of thromboembolism (Geerts et al. 2008). A common characteristic of these studies has been a relatively long hospital stay (6–12 days or more). Since postoperative immobilization is a well-known risk factor for postoperative thromboembolism, the need for prolonged thromboembolic prophylaxis after implementation of fast-track surgery with early mobilization might be questioned. The fast-track group will therefore focus on a large (n = 5,000) prospective, detailed 90-day follow-up study with prophylaxis only until discharge (1–3 days).

It is well-documented that cognitive function declines after THA and TKA in elderly patients (Maze et al. 2008). A fast-track setup may reduce cognitive dysfunction due to less postoperative sleep disturbances, less pain, and less opioid requirements, all of which may impair cognitive function.

Finally, in the fast-track THA and TKA setting, safety aspects will be assessed regarding the risk of hip dislocation, and knee stiffness after TKA.

In summary, despite major improvements in care and short-term outcome after THA and TKA, several issues remain to be answered in order to optimize fast-track treatment.

## References

[CIT0001] Foss NB, Kristensen MT, Jensen PS, Palm H, Krasheninnikoff M, Kehlet H (2009). The effects of liberal versus restrictive transfusion thresholds on ambulation after hip fracture surgery. Transfusion.

[CIT0002] Geerts WH, Bergqvist D, Pineo GF, Heit JA, Samama CM, Lassen MR, Colwell CW (2008). Prevention of venous thromboembolism: American College of Chest Physicians Evidence-Based Clinical Practice Guidelines (8th Edition). Chest.

[CIT0003] Ilfeld BM, Ball ST, Gearen PF, Le LT, Mariano ER, Vandenborne K, Duncan PW, Sessler DI, Enneking FK, Shuster JJ, Theriaque DW, Meyer RS (2008a). Ambulatory continuous posterior lumbar plexus nerve blocks after hip arthroplasty: a dual-center, randomized, triple-masked, placebo-controlled trial. Anesthesiology.

[CIT0004] Ilfeld BM, Le LT, Meyer RS, Mariano ER, Vandenborne K, Duncan PW, Sessler DI, Enneking FK, Shuster JJ, Theriaque DW, Berry LF, Spadoni EH, Gearen PF (2008b). Ambulatory continuous femoral nerve blocks decrease time to discharge readiness after tricompartment total knee arthroplasty: a randomized, triple-masked, placebo-controlled study. Anesthesiology.

[CIT0005] Kehlet H, Wilmore DW (2008). Evidence-based surgical care and the evolution of fast-track surgery. Ann Surg.

[CIT0006] Maze M, Cibelli M, Grocott HP (2008). Taking the lead in research into postoperative cognitive dysfunction. Anesthesiology.

[CIT0007] Minns Lowe CJ, Barker KL, Dewey M, Sackley CM (2007). Effectiveness of physiotherapy exercise after knee arthroplasty for osteoarthritis: systematic review and meta-analysis of randomised controlled trials. BMJ.

[CIT0008] Minns Lowe CJ, Barker KL, Dewey ME, Sackley CM (2009). Effectiveness of physiotherapy exercise following hip arthroplasty for osteoarthritis: a systematic review of clinical trials. BMC Musculoskelet Disord.

[CIT0009] Röstlund T, Kehlet H (2007). High-dose local infiltration analgesia after hip and knee replacement - what is it, why does it work, and what are the future challenges?. Acta Orthop.

